# Physical Exercise−Mediated Changes in Redox Profile Contribute to Muscle Remodeling After Passive Hand-Rolled Cornhusk Cigarette Smoke Exposure

**DOI:** 10.3389/fphys.2020.590962

**Published:** 2020-11-17

**Authors:** Anand Thirupathi, Silvia Scarparo, Paulo L. Silva, Luis F. Marqueze, Franciane T. F. Vasconcelos, Seigo Nagashima, Eduardo B. B. Cunha, Lúcia de Noronha, Paulo C. L. Silveira, Renata T. Nesi, Yaodong Gu, Ricardo A. Pinho

**Affiliations:** ^1^Faculty of Sports Science, Ningbo University, Ningbo, China; ^2^Laboratory of Experimental Pathophysiology, Graduate Program in Health Sciences, Universidade do Extremo Sul Catarinense, Criciúma, Brazil; ^3^Laboratory of Exercise Biochemistry in Health, Graduate Program in Health Sciences, School of Medicine, Pontifícia Universidade Católica do Paraná, Curitiba, Brazil; ^4^Laboratory of Experimental Pathology, Graduate Program in Health Sciences, School of Medicine, Pontifícia Universidade Católica do Paraná, Curitiba, Brazil

**Keywords:** hand-rolled cornhusk cigarette, cigarette smoke, combined physical exercise, oxidative stress, muscle

## Abstract

Consumption of non-traditional cigarettes has increased considerably worldwide, and they can induce skeletal muscle dysfunction. Physical exercise has been demonstrated to be important for prevention and treatment of smoking-related diseases. Therfore, the aim of this study was to investigate the effects of combined physical exercise (aerobic plus resistance exercise) on muscle histoarchitecture and oxidative stress in the animals exposed chronically to smoke from hand-rolled cornhusk cigarette (HRCC). Male Swiss mice were exposed to ambient air or passively to the smoke of 12 cigarettes over three daily sessions (four cigarettes per session) for 30 consecutive days with or without combined physical training. 48 h after the last training session, total leukocyte count was measured in bronchoalveolar lavage fluid (BALF), and the quadriceps were removed for histological/immunohistochemical analysis and measurement of oxidative stress parameters. The effects of HRCC on the number of leukocytes in BALF, muscle fiber diameter, central nuclei, and nuclear factor kappa B (NF-κB) were reverted after combined physical training. In addition, increased myogenic factor 5, tumor necrosis factor alpha (TNFα), reduced transforming growth factor beta (TGF-β), and nitrate levels were observed after physical training. However, the reduction in superoxide dismutase and glutathione/glutathione oxidized ratio induced by HRCC was not affected by the training program. These results suggest the important changes in the skeletal muscle brought about by HRCC-induced alteration in the muscle redox profile. In addition, combined physical exercise contributes to remodeling without disrupting muscle morphology.

## Introduction

Any form of tobacco-containing cigarettes such as industrialized cigarette and handmade cigarette is a complex mixture of toxicants which can pose several risks, such as redox profile alteration and resulting cellular structure deformation in the biological systems (Valença et al., 2005; Gochman et al., 2007; Bhalla et al., 2009; Menegali et al., 2009; Saito et al., 2012; Madani et al., 2018). However, the consumption of other forms of cigarettes, especially handmade or natural cigarettes, which do not undergo industrial processing, has increased considerably worldwide. These cigarettes are erroneously considered healthier than industrialized cigarettes, but the consequences of cigarette smoking are not yet sufficiently well known to generate concern among the public. The use of hand-rolled cornhusk cigarettes (HRCC) has increased in Brazil (Levy et al., 2012), and it has generated intense concern among the public with respect to its effects on health.

Hand-rolled cornhusk cigarette consists of tobacco macerated and rolled up in a corn husk. The effect of this HRCC-exposed smoke is similar to the effect of other types of cigarettes (Valença et al., 2004; Lanzetti et al., 2011; Nesi et al., 2016). However, HRCC smoke can be more severe than other forms of cigarettes because the smoke contains particulate matter and other chemical agents. In addition, the absence of gunpowder in corn straw induces the smoker to exert a more intense inspiratory flow, which leads to a greater uptake of cigarette smoke by the airways when compared to industrialized cigarettes. In fact, these characteristics may potentiate the harmful effects of tobacco and induce alterations in the oxidative stress parameters (Camera et al., 2019). The skeletal muscle is highly susceptible to the effects of cigarette smoke, which can interfere with the quality of muscle structure and function in smokers (Barnes, 2014; Krüger et al., 2018). This cigarette smoke is associated with muscle weakness (Seymour et al., 2010; van den Borst et al., 2011; Barreiro et al., 2012) and reduced muscle mass (Mathur et al., 2014). These effects are attributed to the toxic substances contained in the smoke that stimulates the degradation of muscle proteins and impairs protein synthesis (Rom et al., 2012).

Aerobic physical exercise exerts a protective effect on the oxidative and inflammatory agents present or induced by industrial cigarette smoke (Menegali et al., 2009; Nesi et al., 2016; Madani et al., 2018). Moreover, resistance exercise can also be helpful by increasing muscle tone and density (Saito et al., 2012), by assisting the recovery of respiratory function (Singh et al., 2011), and by stimulating the antioxidant and anti-inflammatory systems (Souza et al., 2017; Vilela et al., 2018). However, the effects of the association of both types of exercise (combined exercise) are still under investigation. Thus, the objective of the present study was to investigate the effects of combined physical exercise on muscle histoarchitecture and oxidative stress parameters in mice exposed to HRCC smoke.

## Materials and Methods

### Animals

Male 3 to 4-month-old Swiss mice (30–35 g) were randomly assigned into four groups (*n* = 9): Ambient air (AA), HRCC, AA-plus combined exercise, and HRCC plus combined exercise. The training protocol and cigarette exposure were performed simultaneously for 4 weeks of experiments. Food and water were available *ad libitum*, and the room temperature was maintained at 20 ± 2°C, with 70% humidity under a 12-h light/dark cycle. The Institutional Committee for Animal Care at Universidade do Extremo Sul Catarinense approved all the procedures under protocol number 087/2015-1.

### HRCC Exposure

Cornhusk cigarettes were purchased from farmers in the city of Severino, RS, Brazil. The tobacco leaves were stripped, macerated, and indirectly exposed to the sun for 48 h. To prepare HRCC, 0.8 g of dry tobacco was uniformly wrapped in a cornhusk. The amount of tobacco used in each cigarette was equivalent to that present in one commercial cigarette. Animals were exposed to 12 HRCC smokes per day; the regimen included exposure to four cigarettes, three times a day, 7 days/week over 30 days (Camera et al., 2019). Briefly, animals were placed in an inhalation chamber (40 cm long, 30 cm wide, and 25 cm high), with an exhaust air system. Each cigarette was connected to a plastic 50-mL syringe, and smoke puffs were aspirated and subsequently injected into the exposure chamber. The animals were maintained under this condition for 6 min. After each cigarette use, the box was opened for 1 min to exhaust the air. The animals were kept in this smoke-air condition for 27 min per session.

### Training Protocol

The combined-exercise training protocol consisted of 30 min of aerobic training and approximately 30 min of resistance training. The training period lasted 4 weeks, and the training frequency was 3–4 days per week, with 48-h intervals between sessions, for a total of 30 days. For aerobic training, the animals were habituated on a ninechannel motor-driven treadmill at 10 m/min for 10 min/day for 1 week to ease their adaptation to the new environment. The mice did not receive any stimuli to run. The exercise groups performed an incremental running program at progressive levels of intensity (13–17 m/min). Untrained animals were placed on a switched-off treadmill for the same 8 weeks as the exercise-trained groups. For resistance training, animals were familiarized with climbing a ladder (1.1 × 0.18 m, 30.2-cm high steps, 80° slope), as previously described (Souza et al., 2017). A load was secured to the base of the tail using plastic insulation tape. A repetition was deemed successful when the animal climbed from the bottom of the rack to the top. The exercise consisted of climbing the ladder carrying a load corresponding to 25% of the animal body weight; the weight was progressively increased to 50 and 75%, with 8–12 repetitions and a 2-min break between repetitions. When necessary, food was placed at the top of the ladder to encourage animals to perform the exercise.

### Bronchoalveolar Lavage (BAL)

The animals were anesthetized with ketamine (150 mg/kg) and xylazine (10 mg/kg), and BAL was performed through a tracheal cannula with 3 × 1 mL phosphate-buffered saline (PBS) lavage. Approximately 1.5 mL (80%) of bronchoalveolar lavage fluid (BALF) was recovered from each mouse examined. A 100 μL aliquot was used for the total cell count, the remainder was immediately centrifuged at 300 × *g* for 10 min, and the cell pellets were washed twice and resuspended. The supernatants of BALF containing total leucocytes were measured in Neubauer counting chamber, and the remaining sample was stored at −80°C.

### Euthanasia and Tissue Preparation

After BAL procedures, all the animals were euthanized by cervical displacement. The right quadriceps (central portion) from three animals from each group were fixed with 4% paraformaldehyde and processed for histology. The remaining samples were aliquoted and stored at −80°C for future biochemical analysis.

### Histological and Morphometrical Analyses

Material cleavage was performed using specific cuts. Formalin fixed paraffin embedded (FFPE) skeletal striated muscle tissue samples were sliced on a microtome to 4-μm-thick histological sections, which were stained with hematoxylin & eosin. For histological analysis, digital images were captured using a slide scanner (AxioScan Zeiss) and diameter was calculated using a software for morphometric analysis (Image Proplus). In each group, 10 photomicrographies were selected in an area of 288815.2563 μm^2^, and the smallest diameter of 10 fibers per field was measured. The data are presented as number and average fiber diameter per field. Quantitative determinations of centralized nuclei were also conducted in the same way as histological analyses using image analysis software. The assessment was performed in a single-blind manner (Stewart et al., 2016).

### Tissue Microarray (TMA) and Immunohistochemistry (IHC)

Representative areas of the muscle were transferred from the histology block to a recipient tissue microarray (TMA) block. Next, two 4-μm-thick paraffin-embedded sections of the TMA blocks were transferred to electrically charged Star Frost^TM^ (Braunschweig, Germany) slides and incubated with a primary anti-nuclear factor kappa B (NF-κB) p105/50 (ab797; 1:200; Abcam, Cambridge, United Kingdom) and Tumor necrosis factor alpha (TNFα) (ab6671; 1:100; Abcam) overnight in a humidified chamber at a temperature between 2 and 8°C. The slides were incubated with the secondary antibody for 30 min at room temperature, using the Reveal Polyvalent horseradish peroxidase (HRP)–Diaminobenzidine (DAB) kit (Detection System-Spring Bioscience^TM^, Pleasanton, CA, United States). The immunoreactivity was developed by adding DAB chromogen/substrate solution (Spring) to the slides. Harris hematoxylin was used for counterstaining. Positive and negative controls were run in parallel with all reactions. The slides were scanned using the Axio Scan.Z1 scanner (Carl Zeiss, Germany). The files generated were fragmented into single images, and approximately 25 images were selected for analysis. The areas of immunopositive markings for the anti-NF-κB p105/50 and TNFα antibodies were quantified using Image-Pro Plus software version 4.5 (Media Cybernetics, United States). The immunopositive objects were selected using a “mask” for standardizing and automating the process. The numerical data of the immunopositive marking area were generated and subsequently exported to an Excel spreadsheet.

### Biochemical Assays

The levels of oxidized intracellular 2′,7′-dichlorofluorescein (DCF) were monitored in samples incubated with 2′,7′-dichlorodihydrofluorescein (DCFH). The formation of the oxidized fluorescent derivative was monitored at excitation and emission wavelengths of 488 and 525 nm, respectively, using fluorescence spectrophotometer instruments (LeBel et al., 1992). To evaluate the indirect nitric oxide (NO) production, the levels of nitrate were measured from of reduction of nitrate by vanadium (III) combined with detection by the acidic Griess reaction (Miranda et al., 2001). Superoxide dismutase (SOD) activity was determined spectrophotometrically at 480 nm and estimated by adrenaline autooxidation inhibition and expressed as U/mg of protein (McCord and Fridovich, 1969). The total glutathione (GSH) levels were based on the reaction of GSH with 5,5′-dithiobis-(2-nitrobenzoic acid) (DTNB) (Ellman’s reagent), which forms an oxidized glutathione (GSSG)–2-nitro-5-thiobenzoic acid (TNB) product that is later reduced by glutathione reductase in the presence of NADPH with the consequent synthesis of GSH. The total GSH concentration was determined using a regression curve that was plotted using various GSH standards. The GSSG level was measured from the recycling of GSSG by the spectrophotometric monitoring of NADPH in the presence of 2-vinylpyridine. The total GSH and GSSG concentrations were determined using a regression curve plotted using various GSH standards (Rahman et al., 2006). The myogenic factor 5 (Myf5) level was measured by western blot. The samples were homogenized in lysis buffer supplemented with protease and phosphatase inhibitors for future total protein extraction. The protein extracts were separated by SDS–PAGE and processed for western blot analysis using antibodies against Myf5 (Santa Cruz Biotechnology, Inc.) according to the standard procedure. The values obtained were normalized to those of β-actin (Santa Cruz Biotechnology). Transforming growth factor beta (TGF-β) tissue concentrations were determined using commercial ELISA kits as recommended by the manufacturer (R&D systems^®^, United States; ALPCO^®^, United States; Labtest^®^, Brazil).

### Statistical Analysis

All data are presented as the mean ± standard error of the mean (SEM), and differences between groups were subjected to one-way analysis of variance (ANOVA) followed by the Newman–Keuls *post hoc* test when appropriate. Differences with *p* < 0.05 were considered statistically significant. All statistical analyses were performed using GraphPad Prism 6 software.

## Results

### HRCC Induces Morphological Alterations in Quadriceps

**FIGURE 1:**
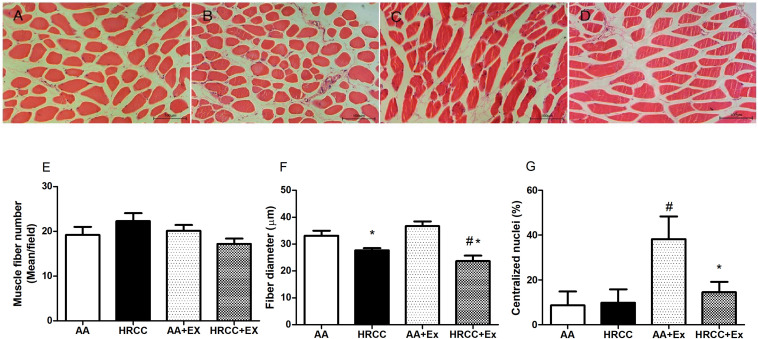
Histology of the quadriceps of mice chronically exposed to hand-rolled cornhusk cigarette (HRCC) smoke and combined exercise. Panels **(A–D)** are photomicrographs stained with H & E: **(A)** ambient air (AA), **(B)** HRCC, **(C)** AA-plus combined exercise, **(D)** HRCC plus combined exercise. Panel **(E)** represents the number of fiber per field, panel **(F)** represents the mean of smallest diameter of 10 fibers per field and panel **(G)** represents the percentage of nuclei centralized. The data are expressed as mean and standard error of the mean and were analyzed statistically using two-way ANOVA, followed by the Newman–Keuls test. The groups were considered different when *p* ≤ 0.05* (difference in relation relation to the ambient air, # difference in relation to HRCC). Images under 20× objective.

Figure 1 shows representative images of cross-sectional histological sections of the quadriceps muscle. Control animals presented a normal morphology and peripheral nuclei muscle fibers (Figure 1A). Animals untrained exposed to HRCC (Figure 1B) exhibited decreased muscle fiber diameter compared to the control animals. In Figure 1C, trained animals showed increased muscle fiber diameter and the presence of the central nuclei. Figure 1D shows a decrease in muscle fiber diameter and the presence of peripheral nuclei on animals exposed to cigarette smoke and combined exercise. These data are represented graphically in Figure 1E (fibers number), Figure 1F (fiber diameter), and Figure 1G (centralized nuclei).

### HRCC Smoke-Induces Myf-5 While Suppressing TGF-β Level

**FIGURE 2:**
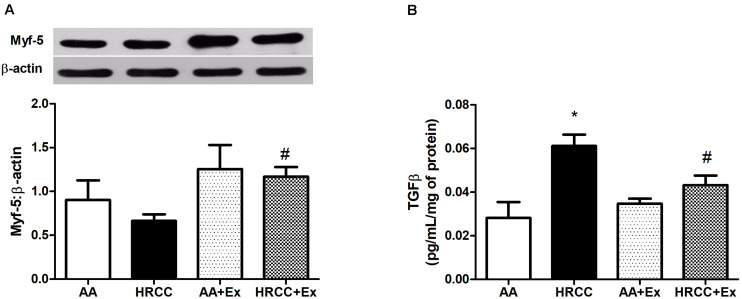
Effect of combined exercise on parameters of muscle remodeling in quadriceps of mice exposed to hand-rolled cornhusk cigarette (HRCC) smoke. The content of myogenic factor 5 (Myf5) **(A)** was normalized to β-actin, and the production of transforming growth factor beta (TGFβ) **(B)** was normalized to the total amount of protein. Data are expressed as mean and standard error of the mean and were analyzed statistically using two-way ANOVA, followed by the Newman–Keuls test. The groups were considered different when *p* ≤ 0.05* (difference in relation to the ambient air, # difference in relation to HRCC).

Combined exercise with HRCC-exposure-induced changes in muscle remodeling. We observed that Myf-5 level was increased in combined training with HRCC-exposed animals when compared to HRCC (Figure 2A), whereas TGF-β level was decreased with combined exercise plus HRCC-exposed animals when compared to HRCC (Figure 2B).

### Combined Physical Exercise Regulates HRCC-Induced Inflammatory Parameters

**FIGURE 3:**
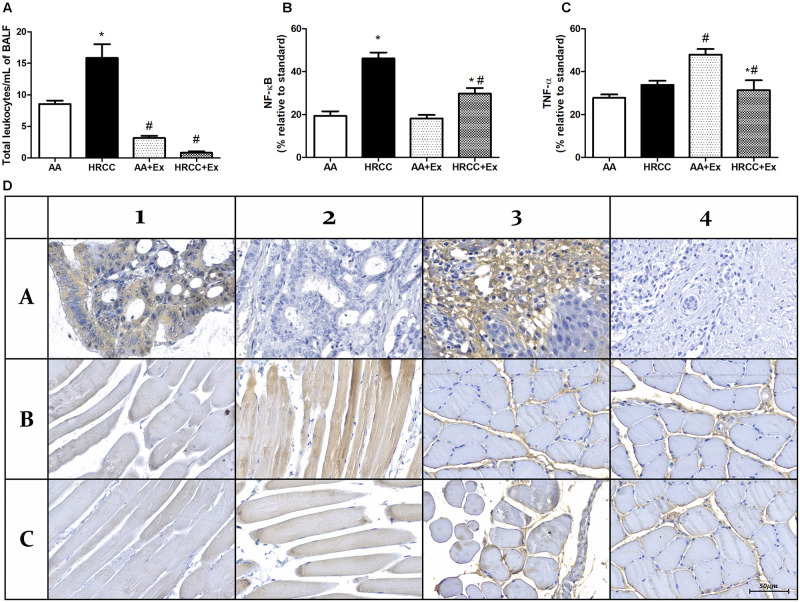
Effect of combined exercise on inflammatory parâmeters of mice exposed to hand-rolled cornhusk cigarette (HRCC) smoke. The number of total leukocytes in Lavage fluid (BALF) **(A)** are expressed as mean and standard error of the mean and were analyzed statistically using two-way ANOVA, followed by the followed by the Newman–Keuls test. The groups were considered different when *p* ≤ 0.05* (difference in relation to the ambient air, # difference in relation to HRCC). Percentage of inflammatory proteins immunoexpression in quadriceps of animals exposed to HRCC and combined exercise were selected from 25 images of four animals per group. The areas of immunopositivity for NF-κB p105/50 **(B)** and TNFα **(C)** were expressed as percentage average relative to standard molecule and analyzed statistically using one-way ANOVA, followed by the Newman–Keuls test. The groups were considered different when the value of *p* was ≤ 0.05* (difference in relation to the ambient air, # difference in relation to HRCC). Representative images of immunohistochemical analysis of quadriceps of animals exposed to HRCC and combined exercise **(D)**. NF-κB positive control (A1), NF-κB negative control (A2), NF-κB Ambient air (B1), NF-κB HRCC (B2), NF-κB AA+exercise (C1) NF-κB HRCC+exercise (C2), (A3), TNFα negative control (A4), TNFα Ambient air (B4), TNFα HRCC (B4), TNFα AA+exercise (C3) TNFα HRCC+exercise (C4).

Figure 3 inflammatory parameters in BALF and quadriceps. The number of total leukocytes in BALF was used as an inflammatory indicator. The results showed an increased number of leukocytes in the animals exposed to HRCC smoke while there was a decrease after physical exercise in the groups of AA+Ex and HRCC+EX (Figure 3A). Quantitative values of immunoexpression of NF-κB p105/50 and TNFα was analyzed and are represented in Figures 3B,C. The immunoexpression of NF-κB p105/50 and TNF (within the muscle fiber) increased in the muscles of animals exposed to HRCC, and it was significantly reduced after physical training. TNFα (in connective tissue) immunoexpression did not show significant changes with HRCC. However, it was significantly increased with exercise. Immunostaining data and representative graphic of these molecules are presented in Figure 3D.

### Combined Exercise Promotes Change in the Oxidative Stress Parameters After HRCC Exposure

**FIGURE 4:**
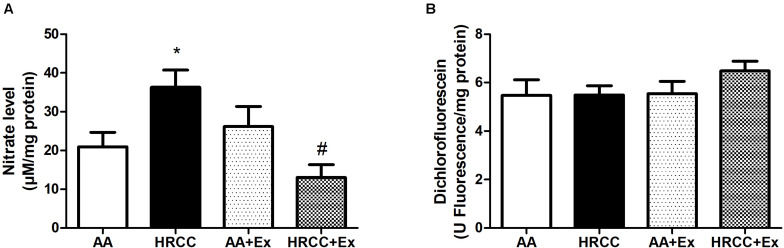
Effect of combined exercise on oxidative stress parameters in quadriceps of mice exposed to hand-rolled cornhusk cigarette (HRCC) smoke. The values of nitric oxide **(A)**, 2′,7′-dichlorofluorescein **(B)** are shown as the mean and standard error of the mean and were analyzed statistically using two-way ANOVA, followed by the Newman–Keuls test. The groups were considered different when *p* ≤ 0.05* (difference in relation to the ambient air, # difference in relation to HRCC).

**FIGURE 5:**
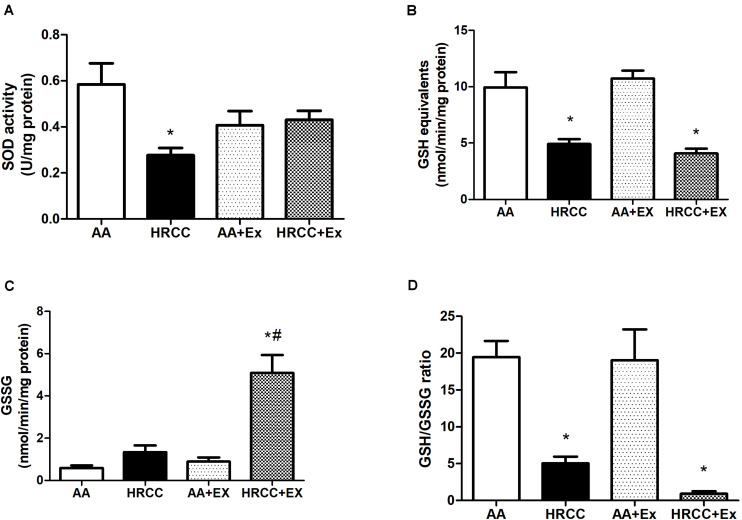
Effect of combined exercise on antioxidants in quadriceps of mice exposed to hand-rolled cornhusk cigarette (HRCC) smoke. Superoxide dismutase activity (SOD) **(A)**, Total GSH activity **(B)**, GSSG level **(C)**, and glutathione (GSH)/oxidized glutathione (GSSG) ratio **(D)** are shown as the mean and standard error of the mean and were analyzed statistically using two-way ANOVA, followed by the Newman–Keuls test. The groups were considered different when *p* ≤ 0.05* (difference in relation to the ambient air, # difference in relation to HRCC).

Levels of DCF and nitrate were used as indicators of cellular oxidants, while the activity of SOD, total GSH, GSSG and the ratio of reduced glutathione to oxidized glutathione (GSH/GSSG) were used as indicators of antioxidant defense systems. The results depicted in Figure 4A show a significant increase in nitrate levels in animals exposed to HRCC smoke and a reduction in this level after combined physical training. However, no significant changes were observed in DCF levels (Figure 4B). SOD activity was significantly reduced with HRCC smoking, but the level of SOD in HRCC with combined exercise program did not have a significant effect (Figure 5A). Total GSH was significantly decreased after HRCC exposure (Figure 5B), while animals of HRCC plus exercise presented increased levels of GSSG when compared to respective controls (Figure 5C). GSH/GSSG ratio was significanlty decreased with HRCC exposure, and these values remained reduced after combined physical training (Figure 5D).

## Discussion

Physical exercise has been widely used to minimize the cigarette-induced damages (Pinho et al., 2007; Menegali et al., 2009; Koubaa et al., 2015; Nesi et al., 2016). Several studies have demonstrated that smokers have muscle abnormalities, which lead to a reduction in exercise performance (Barnes, 2014; Gea et al., 2015). However, studies conducted on cigarette-exposure-induced abnormalities in the skeletal muscle results are inconclusive because of the characteristics of physical exercise and the type of cigarette promote different responses. The effects of exercise are directly dependent on the frequency, intensity, duration, and period of training (Pinho et al., 2006), whereas the type of cigarette and the form of consumption lead possibly to different changes in cardiorespiratory and muscular functions (Camera et al., 2019). In this scenario, this is the first study to show the effects of combined exercise (aerobic plus resistance exercise) on animals’ skeletal muscle exposed to HRCC smoke.

This study showed a decrease in muscle fiber diameter and the presence of peripheral nuclei on animals exposed to HRCC smoke and combined exercise. Previous studies have already demonstrated that chronic smoking induces negative impacts on skeletal muscle structure (Morse et al., 2007; Nogueira et al., 2018), and functions (Degens et al., 2015), while other studies showed improvement in muscle structure in physical exercise (Krüger et al., 2018). When exposed to cigarette smoke, the muscle is more susceptible to atrophy in response to differentiation, proliferation, and remodeling mechanisms (Chan et al., 2020). The muscle morphology alterations induced by HRCC smoke was reverted by combined exercise but, in terms of muscle adaptation, HRCC+exercise group depicts lower fiber diameter and nuclei centralization is not different from HRCC. These results suggest a possible inability of muscle adaptation to combined exercise. This non-adaptive response may be associated with insufficient stimulus (intensity and training period) to induce muscle adaptation mediated by combined exercise. Under these conditions, we observed decreased muscle differentiation by increasing TGFβ expression and inhibiting Myf5 expression. TGFβ is an important marker of tissue remodeling and negatively affects skeletal muscle regeneration by inhibiting satellite cell proliferation and expression of some muscle-specific genes such as Myf-5. Besides, TGFβ is associated with an increase in inflammatory markers (Böhm et al., 2016), and it induces ubiquitin-proteasome protein degradation (Waning et al., 2015). TGFβ reduction by combined exercise may be one of the important mechanisms that contribute to tissue regeneration in the skeletal muscle, especially by stimulating the Akt-mTOR pathway as already demonstrated in smooth muscle cell (Latres et al., 2005; Suwanabol et al., 2012) via IGF-1 signaling, which is stimulated, particularly, through muscle strength exercises. The relationship between TGFβ and Akt-mTOR in the skeletal muscle needs to be better investigated under the effect of cigarette smoking and physical exercise.

Systemic inflammation and oxidative stress are important injury mechanisms that induce independent respiratory and skeletal muscle effects (Cielen et al., 2016). An increase in the recruitment of inflammatory cells to the lung interstitium is observed in smokers (Kennedy-Feitosa et al., 2014), and it presents a risk of tissue damage through the release of toxic mediators, including cytokines, proteolytic enzymes, and ROS (Bhalla et al., 2009). Besides, systemic inflammation is associated with reduced protein synthesis and enhanced protein breakdown, accounting for muscle mass loss and function (Costamagna et al., 2015). In this scenario, our results did not demonstrate alterations in the immunoexpression of TNF-α in muscle tissue after exposure to HRCC. However, exercised animals presented elevated levels of TNF-α, and these results may be related to the intensity of exercise and a possible non-adaptation of the animals to the training protocol. There is no evidence to support the understanding that muscle cells secrete or express TNF-α *in vivo*. Most of the evidence for cytokine expression in the skeletal muscle is derived from the analysis of isolated RNA or protein extracts from the homogenized muscle (Egan and Zierath, 2013; Peake et al., 2015). Therefore, our results revealed immunodetection only in the perisimal area. The elevated level of TNF-α is not correlated only to the grade of muscle inflammation, but also to cellular events during the muscle regeneration in response to injury or non-adaptive processes (Li, 2003).

As presented in this study, previous work has shown that cigarette smoke stimulates the NF-κB activation (Kaisari et al., 2013), and its inhibition prevents muscle degeneration, protein breakdown, and myofiber death (Ahn and Aggarwal, 2005). Although the precise mechanism by which inflammation is involved in protein breakdown/turnover rates is still poorly investigated, Krüger et al. (2018) have recently suggested that a decrease in systemic inflammation and inflammatory mediators in the muscle can indirectly reduce the activation of catabolic pathways via ubiquitin-proteasome proteolytic pathway (Costamagna et al., 2015) and increase anabolic signals (Krüger et al., 2018). Although the contractile muscle activity during exercise also contributes to enhancing the NF-κB levels (Kramer and Goodyear, 2007), moderate exercise training decreases NF-κB activation (Liu and Chang, 2018). Reduced NF-κB levels after exposure to cigarette smoke by exercise may be related to different effects of anti-inflammatory stimulus-induced by moderate exercise, including secretion of anti-inflammatory myokine IL-6, increase in systemic levels of the anti-inflammatory cytokines such as IL-10 and IL-1RA, downregulation of Toll-like receptor expression. Regular exercise lowers circulating numbers of pro-inflammatory monocytes, inhibits monocyte and or macrophage infiltration, and increases Treg cell numbers in circulation (Gleeson et al., 2011; Wang et al., 2020).

**FIGURE 6:**
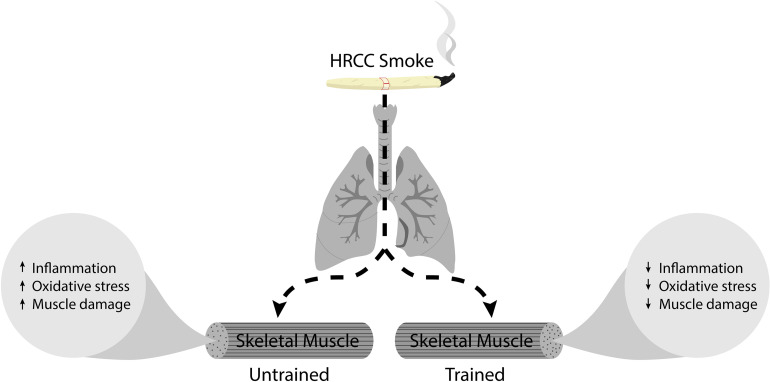
Represents the schematic diagram of HRCC smoke effect on trained and untrained skeletal muscle.

The toxic substances contained in HRCC smoke stimulate the inflammatory response and induce an altered redox system (Figure 6). The skeletal muscle is extremely responsive to cigarette smoke (Barnes, 2014; Krüger et al., 2018), favoring ROS production. Here, no significant alteration in the DCF production was observed in the different experimental groups, However, nitrate levels were significantly affected by both HRCC smoke and combined exercise. The results about DCF are apparently surprising because previous studies have already demonstrated that cigarette smoke induces elevated levels of DCF in the lung that are reduced after physical training (Menegali et al., 2009; Nesi et al., 2016). However, the skeletal muscle seems to respond differently to cigarette stimulus than the lung. Observed changes in nitrate levels from HRCC exposure, suggest a possible participation of reactive species of nitrogen on the redox system of the skeletal muscle. This can be reinforced by the low activity of SOD, and the concomitant increase in nitrate levels suggests an alternative pathway to dismutation of superoxide leading to the formation of peroxynitrite (Jourd’heuil et al., 2001). After a combined physical training program, a reduced level of nitrate was observed as well as an increase in SOD activity without changing the DCF levels. This response from exercise is possibly associated with the effects of exercise on the activity of enzymes that catalyze hydrogen peroxide (Pinho et al., 2006; Scheffer et al., 2012; Tromm et al., 2016).

Cigarette-induced glutathione system depletion has been revealed in previous studies (Raza et al., 2013; Gould et al., 2015). Our results show that after exposure to HRCC smoke presented a lower level of total GSH and GSH/GSSG ratio than in control, and this effect was more accentuated with physical exercise. Previous studies have already shown that under the influence of cigarette smoke, the GSH/GSSG ratio is reduced in plasma (Moriarty et al., 2003), heart, and liver (Montiel-Duarte et al., 2004), and other studies indicate a reduced level of GSH in lung (Camera et al., 2019). In skeletal muscle, GSH levels vary depending on the metabolic profile of the tissue (Sen, 1998) and the state of physical exercise (Ji et al., 1992). Notably, the results of this study show that physical training further decreases the cigarette-induced GSH/GSSG ratio. This may be associated with increased detoxification induced by physical exercise. Physical exercise increases the expression and activity of glutathione peroxidase (GPx) in muscle (Lambertucci et al., 2007; Nguyen et al., 2016). GPx catalyzes the reduction of organic hydroperoxides using GSH as a reductant (Brigelius-Flohé and Maiorino, 2013). If the resynthesis of GSH is impaired or if the oxidation rate is greater than resynthesis under the effect of HRCC cigarettes, the level of GSSG remains high, reducing the GSH/GSSG ratio (Lambertucci et al., 2007).

## Limitation of the Study

This study selected only male mice to investigate the effect of combined physical exercise that were exposed to AA or passively to the smoke HRCC. This could limit the outcome of precise prediction of sex differences in the redox profile that contribute muscle remodeling after the smoke exposure. Although, studies have shown that female gender have type II fiber atrophy and greater loss of muscle strength when compared to male gender, indicating that intrinsic properties of muscle are prone to altered in female smokers, and this effect is more related to their amount of physical activity (Ausín et al., 2017). Regarding muscle structure with cigarette smoking people, percentage of type II fibers lower in female than male even in similar nutritional status, systemic inflammation, and physical activity (Gosker et al., 2007). Ausín et al. (2017) showed that female with cigarette smoking history had higher level of muscle damage than male. But males showed improved signs of early steps of muscle regeneration than cigarette smoking females. However, these findings suggest that different susceptibility of patients of both genders to injury may be linked with different stimuli such as tobacco and/or amount of physical activity. Furthermore, the earlier muscle regeneration capacity with male cigarette smoking does not necessarily means that it can complete its reparative process will continue correctly until the end (Thériault et al., 2014). Other factors like vitamin D deficiency is positively influenced with the muscle mass, and female with smoking history is more pronounced with vitamin D deficiency (Dawson-Hughes, 2012). In contrast, Sharanya et al. (2019), observed that female gender who have smoking history and longer times spent standing have reduced muscle fiber size, muscle strength and peak workload. However, this sex difference occurred due to increased circulating pro-inflammatory cytokines in female compared to male. However, this study did not find direct correlations between increased markers of systemic inflammation and fiber atrophy, nor indeed between physical activity levels and fiber atrophy in either sex. This suggests that any contribution of systemic inflammation or physical activity to fiber atrophy is moderated by other influences. Moreover, diffusion abnormalities develop earlier in female than male that causes muscle atrophy and weakness, and this may be due to differences in aetiological factors and downstream signaling pathways that affect the skeletal muscle structure and function (Sharanya et al., 2019). Regarding aging, increasing age is not able to maintain organ integrity. Consequently, less protective against oxidative damage. Recent report have shown that maintenance of peripheral muscle mass in cigarette smoking people is compromised with accelerated aging, and the accelerated aging phenotype is a result of DNA repair impairment and dysregulation of cellular homeostasis in the muscle of cigarette smoking people (Lakhdar et al., 2018). Further, studies have revealed that men and women with cigarette smoking have structural and functional differences in the peripheral muscles (Ausín et al., 2017). Taken together, these studies suggest that females who have smoking history have greater prevalence of muscle damage and weakness than males, suggesting that sex and age influence muscle phenotype and function in smoking people.

## Conclusion

These results suggest that HRCC smoke induces important morphological changes in the skeletal muscle by altering the muscle redox profile, and it may impair muscle function. In contrast, combined exercise plays an important role in the remodeling process, but its effect is dependent on the response of the redox system.

## Data Availability Statement

The raw data supporting the conclusions of this article will be made available by the authors, without undue reservation.

## Ethics Statement

The Institutional Committee for Animal Care at Universidade do Extremo Sul Catarinense approved all the procedures under protocol number 087/2015-1.

## Author Contributions

AT, SS, PS, LM, FV, RN, and SN conceived the presented idea, developed the framework, and wrote the manuscript. AT, EC, LN, PCS, YG, and RP provided critical feedback and contributed to the final version. All authors were involved in the final direction of the manuscript, contributed to the final version of the manuscript, and have read and agreed to the published version of the manuscript.

## Conflict of Interest

The authors declare that the research was conducted in the absence of any commercial or financial relationships that could be construed as a potential conflict of interest.

## References

[B1] Ahn K. S. Aggarwal B. B. (2005). Transcription factor NF-kappaB: a sensor for smoke and stress signals. *Ann. N. Y. Acad. Sci.* 1056 218–233. 10.1196/annals.1352.026 16387690

[B2] Ausín P. Martínez-Llorens J. Sabaté-Bresco M. Casadevall C. Barreiro E. Gea J. (2017). Sex differences in function and structure of the quadriceps muscle in chronic obstructive pulmonary disease patients. *Chron. Respir. Dis.* 14 127–139. 10.1177/1479972316674412 27923983 PMC5720222

[B3] Barnes P. J. (2014). Cellular and molecular mechanisms of chronic obstructive pulmonary disease. *Clin. Chest Med.* 35 71–86. 24507838 10.1016/j.ccm.2013.10.004

[B4] Barreiro E. del Puerto-Nevado L. Puig-Vilanova E. Pérez-Rial S. Sánchez F. Martínez-Galán L. (2012). Cigarette smoke-induced oxidative stress in skeletal muscles of mice. *Respir. Physiol. Neurobiol.* 182 9–17. 10.1016/j.resp.2012.02.001 22349133

[B5] Bhalla D. K. Hirata F. Rishi A. K. Gairola C. G. (2009). Cigarette smoke, inflammation, and lung injury: a mechanistic perspective. *J. Toxicol. Environ. Health. B Crit. Rev.* 12 45–64. 10.1080/10937400802545094 19117209

[B6] Böhm A. Hoffmann C. Irmler M. Schneeweiss P. Schnauder G. Sailer C. (2016). TGF-β contributes to impaired exercise response by suppression of mitochondrial key regulators in skeletal muscle. *Diabetes* 65 2849–2861. 10.2337/db15-1723 27358493

[B7] Brigelius-Flohé R. Maiorino M. (2013). Glutathione peroxidases. *Biochim. Biophys. Acta.* 1830 3289–303. 10.1016/j.bbagen.2012.11.020 23201771

[B8] Camera F. D. Pozzi B. G. Paganini C. de S. Sorato H. R. Tavares F. (2019). Cardioprotective effects of physical exercise on redox biology in mice exposed to hand-rolled cornhusk cigarette smoke. *Arch. Biochem. Biophys.* 661 50–55. 10.1016/j.abb.2018.11.003 30414729

[B9] Chan S. M. H. Cerni C. Passey S. Seow H. J. Bernardo I. van der Poel C. (2020). Cigarette smoking exacerbates skeletal muscle injury without compromising its regenerative capacity. *Am. J. Respir. Cell. Mol. Biol.* 62 217–230. 10.1165/rcmb.2019-0106oc 31461300

[B10] Cielen N. Maes K. Heulens N. Troosters T. Carmeliet G. Janssens W. (2016). Interaction between physical activity and smoking on lung, muscle, and bone in mice. *Am. J. Respir. Cell Mol. Biol.* 54 674–682. 10.1165/rcmb.2015-0181oc 26448063

[B11] Costamagna D. Costelli P. Sampaolesi M. Penna F. (2015). Role of inflammation in muscle homeostasis and myogenesis. *Mediat. Inflamm.* 2015:805172. 10.1155/2015/805172 PMC4609834 26508819

[B12] Dawson-Hughes B. (2012). Serum 25-hydroxyvitamin D and muscle atrophy in the elderly. *Proc. Nutr. Soc.* 71 46–49. 10.1017/s0029665111003260 22040926

[B13] Degens H. Gayan-Ramirez G. van Hees H. W. (2015). Smoking-induced skeletal muscle dysfunction: from evidence to mechanisms. *Am. J. Respir. Crit. Care Med.* 191 620–625. 10.1164/rccm.201410-1830pp 25581779

[B14] Egan B. Zierath J. R. (2013). Exercise metabolism and the molecular regulation of skeletal muscle adaptation. *Cell Metab.* 17 162–184. 10.1016/j.cmet.2012.12.012 23395166

[B15] Gea J. Pascual S. Casadevall C. Orozco-Levi M. Barreiro E. (2015). Muscle dysfunction in chronic obstructive pulmonary disease: update on causes and biological findings. *J. Thorac. Dis.* 7 E418–E438. 26623119 10.3978/j.issn.2072-1439.2015.08.04 PMC4635259

[B16] Gleeson M. Bishop N. C. Stensel D. J. Lindley M. R. Mastana S. S. Nimmo M. A. (2011). The anti-inflammatory effects of exercise: mechanisms and implications for the prevention and treatment of disease. *Nat. Rev. Immunol.* 11 607–615. 10.1038/nri3041 21818123

[B17] Gochman E. Reznick A. Z. Avizohar O. Ben-Amotz A. Levy Y. (2007). Exhaustive exercise modifies oxidative stress in smoking subjects. *Am. J. Med. Sci.* 333 346–353. 10.1097/maj.0b013e318065b57c 17570987

[B18] Gosker H. R. Zeegers M. P. Wouters E. F. Schols A. M. (2007). Muscle fibre type shifting in the vastus lateralis of patients with COPD is associated with disease severity: a systematic review and meta-analysis. *Thorax* 62 944–949. 10.1136/thx.2007.078980 17526675 PMC2117111

[B19] Gould N. S. Min E. Huang J. Chu H. W. Good J. Martin R. J. (2015). Glutathione depletion accelerates cigarette smoke-induced inflammation and airspace enlargement. *Toxicol. Sci.* 147 466–474. 10.1093/toxsci/kfv143 26149495 PMC4707200

[B20] Ji L. L. Fu R. Mitchell E. W. (1992). Glutathione and antioxidant enzymes in skeletal muscle: effects of fiber type and exercise intensity. *J. Appl. Physiol.* 73 1854–1859. 10.1152/jappl.1992.73.5.1854 1474061

[B21] Jourd’heuil D. Jourd’heuil F. L. Kutchukian P. S. Musah R. A. Wink D. A. Grisham M. B. (2001). Reaction of superoxide and nitric oxide with peroxynitrite. Implications for peroxynitrite-mediated oxidation reactions in vivo. *J. Biol. Chem.* 276 28799–28805. 10.1074/jbc.m102341200 11373284

[B22] Kaisari S. Rom O. Aizenbud D. Reznick A. Z. (2013). Involvement of NF-κB and muscle specific E3 ubiquitin ligase MuRF1 in cigarette smoke-induced catabolism in C2 myotubes. *Adv. Exp. Med. Biol.* 788 7–17. 10.1007/978-94-007-6627-3_2 23835952

[B23] Kennedy-Feitosa E. Pinto R. F. S. Pires K. M. P. Monteiro A. P. T. Machado M. N. Santos J. C. (2014). The influence of 5-lipoxygenase on cigarette smoke-induced emphysema in mice. *Biochim. Biophys. Acta Gen. Subj.* 1840 199–208. 10.1016/j.bbagen.2013.09.028 24076233

[B24] Koubaa A. Triki M. Trabelsi H. Masmoudi L. Zeghal K. N. Sahnoun Z. (2015). Effect of low-intensity continuous training on lung function and cardiorespiratory fitness in both cigarette and hookah smokers. *Afr. Health Sci.* 15 1170–1181. 10.4314/ahs.v15i4.16 26958018 PMC4765424

[B25] Kramer H. F. Goodyear L. J. (2007). Exercise, MAPK, and NF-kappaB signaling in skeletal muscle. *J. Appl. Physiol*. 103 388–395. 10.1152/japplphysiol.00085.2007 17303713

[B26] Krüger K. Seimetz M. Ringseis R. Wilhelm J. Pichl A. Couturier A. (2018). Exercise training reverses inflammation and muscle wasting after tobacco smoke exposure. *Am. J. Physiol. Regul. Integr. Comp. Physiol.* 314 R366–R376. 29092860 10.1152/ajpregu.00316.2017

[B27] Lakhdar R. McGuinness D. Drost E. M. Shiels P. G. Bastos R. MacNee W. (2018). Role of accelerated aging in limb muscle wasting of patients with COPD. *Int. J. Chron. Obstruct. Pulmon. Dis.* 13 1987–1998. 10.2147/copd.s155952 29970961 PMC6022820

[B28] Lambertucci R. H. Levada-Pires A. C. Rossoni L. V. Curi R. Pithon-Curi T. C. (2007). Effects of aerobic exercise training on antioxidant enzyme activities and mRNA levels in soleus muscle from young and aged rats. *Mech. Ageing Dev.* 128 267–275. 10.1016/j.mad.2006.12.006 17224177

[B29] Lanzetti M. Lopes A. A. Ferreira T. S. de Moura R. S. Resende A. C. Porto L. C. (2011). Mate tea ameliorates emphysema in cigarette smoke-exposed mice. *Exp. Lung Res.* 37 246–257. 10.3109/01902148.2010.535092 21210748

[B30] Latres E. Amini A. R. Amini A. A. Griffiths J. Martin F. J. Wei Y. (2005). Insulin-like growth factor-1 (IGF-1) inversely regulates atrophy-induced genes via the phosphatidylinositol 3-kinase/Akt/mammalian target of rapamycin (PI3K/Akt/mTOR) pathway. *J. Biol. Chem.* 280 2737–2744. 10.1074/jbc.m407517200 15550386

[B31] LeBel C. P. Ischiropoulos H. Bondy S. C. (1992). Evaluation of the probe 2’,7’-dichlorofluorescin as an indicator of reactive oxygen species formation and oxidative stress. *Chem. Res. Toxicol.* 5 227–231. 10.1021/tx00026a012 1322737

[B32] Levy D. de Almeida L. M. Szklo A. (2012). The Brazil simsmoke policy simulation model: the effect of strong tobacco control policies on smoking prevalence and smoking-attributable deaths in a middle income nation. *PLoS Med.* 9:e1001336. 10.1371/journal.pmed.1001336 23139643 PMC3491001

[B33] Li Y. P. (2003). TNF-alpha is a mitogen in skeletal muscle. *Am. J. Physiol. Cell. Physiol.* 285 C370–C376. 12711593 10.1152/ajpcell.00453.2002

[B34] Liu H. W. Chang S. J. (2018). Moderate exercise suppresses NF-κB signaling and activates the SIRT1-AMPK-PGC1α axis to attenuate muscle loss in diabetic db/db Mice. *Front. Physiol.* 9:636. 10.3389/fphys.2018.00636 29896118 PMC5987703

[B35] Madani A. Alack K. Richter M. J. Krüger K. (2018). Immune-regulating effects of exercise on cigarette smoke-induced inflammation. *J. Inflamm. Res.* 11 155–167. 10.2147/jir.s141149 29731655 PMC5923223

[B36] Mathur S. Brooks D. Carvalho C. R. F. (2014). Structural alterations of skeletal muscle in copd. *Front. Physiol.* 5:104. 10.3389/fphys.2014.00104 24678302 PMC3958732

[B37] McCord J. M. Fridovich I. (1969). Superoxide dismutase. An enzymic function for erythrocuprein (hemocuprein). *J. Biol. Chem.* 244 6049–6055. 5389100

[B38] Menegali B. T. Nesi R. T. Souza P. S. Silva L. A. Silveira P. C. L. Valença S. S. (2009). The effects of physical exercise on the cigarette smoke-induced pulmonary oxidative response. *Pulm. Pharmacol. Ther.* 22 567–573. 10.1016/j.pupt.2009.08.003 19683592

[B39] Miranda K. M. Espey M. G. Wink D. A. (2001). A rapid, simple spectrophotometric method for simultaneous detection of nitrate and nitrite. *Nitr. Oxide* 5 62–71. 10.1006/niox.2000.0319 11178938

[B40] Montiel-Duarte C. Ansorena E. López-Zabalza M. J. Cenarruzabeitia E. Iraburu M. J. (2004). Role of reactive oxygen species, glutathione and NF-kappaB in apoptosis induced by 3,4-methylenedioxymethamphetamine (“Ecstasy”) on hepatic stellate cells. *Biochem. Pharmacol.* 67 1025–1033. 10.1016/j.bcp.2003.10.020 15006539

[B41] Moriarty S. E. Shah J. H. Lynn M. Jiang S. Openo K. Jones D. P. (2003). Oxidation of glutathione and cysteine in human plasma associated with smoking. *Free Radic. Biol. Med.* 35 1582–1588. 10.1016/j.freeradbiomed.2003.09.006 14680681

[B42] Morse C. I. Wüst R. C. Jones D. A. de Haan A. Degens H. (2007). Muscle fatigue resistance during stimulated contractions is reduced in young male smokers. *Acta Physiol.* 191 123–129. 10.1111/j.1748-1716.2007.01721.x 17550408

[B43] Nesi R. T. de Souza P. S. dos Santos G. P. Thirupathi A. Menegali B. T. Silveira P. C. L. (2016). Physical exercise is effective in preventing cigarette smoke-induced pulmonary oxidative response in mice. *Int. J. Chron. Obstruct. Pulmon. Dis.* 11 603–610. 10.2147/copd.s93958 27042047 PMC4809330

[B44] Nguyen A. Duquette N. Mamarbachi M. Thorin E. (2016). Epigenetic regulatory effect of exercise on glutathione peroxidase 1 expression in the skeletal muscle of severely dyslipidemic mice. *PLoS One* 11:e0151526. 10.1371/journal.pone.0151526 27010651 PMC4806847

[B45] Nogueira L. Trisko B. M. Lima-Rosa F. L. Jackson J. Lund-Palau H. Yamaguchi M. (2018). Cigarette smoke directly impairs skeletal muscle function through capillary regression and altered myofibre calcium kinetics in mice. *J. Physiol.* 596 2901–2916. 10.1113/jp275888 29797443 PMC6046067

[B46] Peake J. M. Della Gatta P. Suzuki K. Nieman D. C. (2015). Cytokine expression and secretion by skeletal muscles:regulatory mechanisms and exercise effects. *Exerc. Immunol. Rev.* 21 8–25. 25826432

[B47] Pinho R. A. Andrades M. E. Oliveira M. R. Pirola A. C. Zago M. S. Silveira P. C. L. (2006). Imbalance in SOD/CAT activities in rat skeletal muscles submitted to treadmill training exercise. *Cell Biol. Int.* 30 848–853. 10.1016/j.cellbi.2006.03.011 17011801

[B48] Pinho R. A. Chiesa D. Mezzomo K. M. Andrades M. E. Bonatto F. Gelain D. (2007). Oxidative stress in chronic obstructive pulmonary disease patients submitted to a rehabilitation program. *Respir. Med.* 101 1830–1835. 10.1016/j.rmed.2007.02.004 17376663

[B49] Rahman I. Kode A. Biswas S. K. (2006). Assay for quantitative determination of glutathione and glutathione disulfide levels using enzymatic recycling method. *Nat. Protoc.* 1 3159–3165. 10.1038/nprot.2006.378 17406579

[B50] Raza H. John A. Nemmar A. (2013). Short-term effects of nose-only cigarette smoke exposure on glutathione redox homeostasis, cytochrome P450 1A1/2 and respiratory enzyme activities in mice tissues. *Cell. Physiol. Biochem.* 31 683–692. 10.1159/000350087 23711494

[B51] Rom O. Kaisari S. Aizenbud D. Reznick A. Z. (2012). Identification of possible cigarette smoke constituents responsible for muscle catabolism. *J. Muscle Res. Cell Motil.* 33 199–208. 10.1007/s10974-012-9299-4 22614737

[B52] Saito T. Miyatake N. Sakano N. Oda K. Katayama A. Nishii K. (2012). Relationship between cigarette smoking and muscle strength in Japanese men. *J. Prev. Med. Public Heal.* 45 381–386. 10.3961/jpmph.2012.45.6.381 23230468 PMC3514468

[B53] Scheffer D. L. Silva L. A. Tromm C. B. da Rosa G. L. Silveira P. C. L. de Souza C. T. (2012). Impact of different resistance training protocols on muscular oxidative stress parameters. *Appl. Physiol. Nutr. Metab.* 37 1239–1246. 10.1139/h2012-115 23176530

[B54] Sen C. K. (1998). “Glutathione: a key role in skeletal muscle metabolism,” in *Oxidative Stress in Skeletal Muscle. MCBU Molecular and Cell Biology Updates*, eds Reznick A. Z. Packer L. Sen C. K. Holloszy J. O. Jackson M. J. (Basel: Birkhäuser). 10.1007/978-3-0348-8958-2_8

[B55] Seymour J. M. Spruit M. A. Hopkinson N. S. Natanek S. A. Man W. D.-C. Jackson A. (2010). The prevalence of quadriceps weakness in COPD and the relationship with disease severity. *Eur. Respir. J.* 36 81–88. 10.1183/09031936.00104909 19897554 PMC3039205

[B56] Sharanya A. Ciano M. Withana S. Kemp P. R. Polkey M. I. Sathyapala S. A. (2019). Sex differences in COPD-related quadriceps muscle dysfunction and fibre abnormalities. *Chron. Respir. Dis.* 16 1479973119843650. 10.1177/1479973119843650 PMC6537500 31131626

[B57] Singh V. P. Jani H. John V. Singh P. Joseley T. (2011). Effects of upper body resistance training on pulmonary functions in sedentary male smokers. *Lung India* 28 169–173. 10.4103/0970-2113.83971 21886949 PMC3162752

[B58] Souza P. S. Gonçalves E. D. Pedroso G. S. Farias H. R. Junqueira S. C. Marcon R. (2017). Physical exercise attenuates experimental autoimmune encephalomyelitis by inhibiting peripheral immune response and blood-brain barrier disruption. *Mol. Neurobiol.* 54 4723–4737. 10.1007/s12035-016-0014-0 27447807

[B59] Stewart M. D. Lopez S. Nagandla H. Soibam B. Benham A. Nguyen J. (2016). Mouse myofibers lacking the SMYD1 methyltransferase are susceptible to atrophy, internalization of nuclei and myofibrillar disarray. *Dis. Model. Mech.* 9 347–359. 10.1242/dmm.022491 26935107 PMC4833328

[B60] Suwanabol P. A. Seedial S. M. Zhang F. Shi X. Si Y. Liu B. (2012). TGF-β and Smad3 modulate PI3K/Akt signaling pathway in vascular smooth muscle cells. *Am. J. Physiol. Circ. Physiol.* 302 H2211–H2219. 10.1152/ajpheart.00966.2011 PMC3378292 22447946

[B61] Thériault M. E. Paré M. È Lemire B. B. Maltais F. Debigaré R. (2014). Regenerative defect in vastus lateralis muscle of patients with chronic obstructive pulmonary disease. *Respir. Res.* 25 15–35. 10.1186/1465-9921-15-35 PMC3987676 24666540

[B62] Tromm C. B. Pozzi B. G. Paganini C. S. Marques S. O. Pedroso G. S. Souza P. S. (2016). The role of continuous versus fractionated physical training on muscle oxidative stress parameters and calcium-handling proteins in aged rats. *Aging Clin. Exp. Res.* 28 833–841. 10.1007/s40520-015-0501-6 26620674

[B63] Valença S. S. da Hora K. Castro P. Moraes V. G. Carvalho L. Porto L. C. (2004). Emphysema and metalloelastase expression in mouse lung induced by cigarette smoke. *Toxicol. Pathol.* 32 351–356. 10.1080/01926230490431466 15204978

[B64] Valença S. S. Lima E. A. C. Dire G. F. Bernardo-Filho M. Porto L. C. (2005). Sodium pertechnetate (Na99mTcO_4_) biodistribution in mice exposed to cigarette smoke. *BMC Nucl. Med.* 5:1. 10.1186/1471-2385-5-1 15823206 PMC1090589

[B65] van den Borst B. Koster A. Yu B. Gosker H. R. Meibohm B. Bauer D. C. (2011). Is age-related decline in lean mass and physical function accelerated by obstructive lung disease or smoking? *Thorax* 66 961–969. 10.1136/thoraxjnl-2011-200010 21724748 PMC3285455

[B66] Vilela T. C. Effting P. S. dos Santos, Pedroso G. Farias H. Paganini L. (2018). Aerobic and strength training induce changes in oxidative stress parameters and elicit modifications of various cellular components in skeletal muscle of aged rats. *Exp. Gerontol.* 106 21–27. 10.1016/j.exger.2018.02.014 29471131

[B67] Wang J. Liu S. Li G. Xiao J. (2020). Exercise regulates the immune system. *Adv. Exp. Med. Biol.* 1228 395–408. 10.1007/978-981-15-1792-1_27 32342473

[B68] Waning D. L. Mohammad K. S. Reiken S. Xie W. Andersson D. C. John S. (2015). Excess TGF-β mediates muscle weakness associated with bone metastases in mice. *Nat. Med.* 21 1262–1271. 10.1038/nm.3961 26457758 PMC4636436

